# The American Society for Physicians’ Study Travels

**Published:** 1913-11

**Authors:** Albert Bernheim

**Affiliations:** Secretary-General


### THE AMERICAN SOCIETY FOR PHYSICIANS' STUDY TRAVELS.

Presidents: Dr. Jas. M. Anders, Philadelphia, Pa.; Dr. Wm. J. Mayo, Rochester, Minn.; Dr. Lewellys F. Barker, Baltimore, Md.; Dr. Rudolph Matas, New Orleans, La.

Secretary General: Dr. Albert Bernheim, Philadelphia, Pa.

Department Directors: Finance, Dr. L. Webster Fox, Travel Recorder, vacant; Publicity, Dr. Alfred Stengel; Postgraduate Work, vacant; Travel Manager, Dr. E. E. Montgomery.

Philadelphia, Pa., U. S. A.
1225 Spruce Street,
October 21st, 1913.

Dr. R. M. Harbin, Secretary-Treasurer,
Georgia Surgeons' Club,
Rome, Ga.

Dear Doctor Harbin:

In answer to your kind letter of the 18th inst., I am sending you a copy of our Constitution and By-laws, a copy of our first list of the members of Two Hundred Committee, a General Committee comprising the names of physicians residing all over the country. Since this list was printed, many other names have been added, and many physicians have joined. I also enclose a letter of invitation to become member in our Society, and application card. We have patterned our Society after similar societies existing in Germany and France. You undoubtedly know that the German Society visited America last year, which visit was the fourteenth study travel tour of the German Society. We intend to undertake every year at least one travel tour. In our meeting of the Executive Committee next week, we will definitely decide upon the tour for 1914. This tour very probably will be subsequent to the Atlantic City meeting of the A. M. A., and will take in Northern Pennsylvania, New York, Eastern Canada, and New England States, with stops at various places and cities where the Society may be able to see and learn something, i. e. in the nature of a postgradute course (lectures and demonstrations) and of course recreation will not be forgotten. Definite plans as to date, places, program, and expenses will be sent out in the very near future.

Any future data will be gladly given to you. We should be very glad to have you join our Society. I certainly should appreciate, if your Georgia Surgeons' Club could see its way to join hands with us. Last February I sent out a number of letters also to Georgia, to ask colleagues to act as members of the Committee of Two Hundred.

Trusting to hear from you soon again, I am,

Yours very truly,

ALBERT BERNHEIM,

Secretary-General.

Copy referred to the Committeemen.

---

Melted vaseline is an important adjunct in the transfusion paraphernalia. Frequently applied at the site of junction, it minimizes any tendency to clot formation.

As a quick method of cauterizing the appendix stump, amputate the organ with knife or scissors dipped in pure phenol.

In case of intractable "dyspepsia" persistent tenderness in the right iliac region is suggestive of chronic appendicitis as the cause.

In edema of the lungs of cardiac origin a small dose of morphine often does more good than all the simulants. It may be the only treatment needed.—American Journal of Surgery.

